# Case Report: Paradoxical Inflammatory Response Syndrome in a Previously Healthy, HIV-Negative, Pediatric Patient With *Cryptococcus gatii* Meningitis

**DOI:** 10.3389/fped.2021.703895

**Published:** 2021-08-25

**Authors:** Jessica H. Cheng, Ritu Cheema, Peter R. Williamson, Victoria R. Dimitriades

**Affiliations:** ^1^Department of Pediatrics, UC Davis Health, Sacramento, CA, United States; ^2^Division of Pediatric Infectious Diseases, Department of Pediatrics, UC Davis Health, Sacramento, CA, United States; ^3^Laboratory of Clinical Immunology and Microbiology, National Institute of Allergy and Infectious Diseases (NIAID), Bethesda, MD, United States; ^4^Division of Pediatric Allergy, Immunology & Rheumatology, Department of Pediatrics, UC Davis Health, Sacramento, CA, United States

**Keywords:** cryptococcal meningitis, post-infectious inflammatory response syndrome, immunocompetent, pediatrics, IL-6

## Abstract

The immunological response of patients with cryptococcal meningitis (CM), particularly those not known to be immunocompromised, has generated an increased interest recently. Although CM is an infection with significant rates of morbidity and mortality, its sequelae may also include a post-infectious inflammatory response syndrome (PIIRS) in patients who have already achieved microbiological control. PIIRS can cause substantial immune-mediated damage to the central nervous system resulting in long-term neurological disability or even death. Steroids have been used successfully in the management of PIIRS in adults. In this report, we present the case of a previously healthy adolescent male with *Cryptococcus gattii* meningitis who experienced neurological deterioration due to PIIRS after the initiation of antifungal therapy. Immunological workup did not demonstrate any frank underlying immunodeficiencies, and genetic primary immunodeficiency screening was unremarkable. He was treated with steroids and recovered clinically; however, intermittent inflammatory episodes needed to be managed through several flares of symptoms. In the setting of the current literature, we discuss the management and monitoring of PIIRS in a pediatric patient, along with considerations of targeted future therapies.

## Introduction

Cryptococcal meningitis (CM) causes significant morbidity and mortality. While previously found to be most common in HIV-positive individuals, since the widespread use of antiretroviral therapy (ART) in these patients, the HIV-negative, previously healthy individuals have become an increasingly large subset of those with CM (1). A national epidemiological study of hospitalizations for CM over a 13-year period demonstrated that hospitalizations in the HIV-positive population were decreasing, while hospitalizations in the HIV-negative population remained relatively stable (1). Although in-hospital mortality decreased over the study period, it was still significant at 12.4% for women and 10.8% for men. Data are more limited in the pediatric population. One study of hospital admissions for cryptococcal infections in a large national pediatric database showed 6.2 cases per million hospitalizations with 21% of these cases occurring in immunocompetent patients and 38% of those cases involving the central nervous system (2). Of the patients with CM, 20.8% underwent neurosurgical procedures, and 50% received mechanical ventilation; in-hospital mortality rate was 21.5%. In a retrospective review of pediatric HIV-negative cryptococcal meningitis cases at a major tertiary care center, only 23.5% were found to have identifiable underlying disease, with treatment failure occurring in 35.3% of the patients (3).

Recent detailed immunological studies in HIV-negative and previously healthy individuals have been crucial to the understanding of the severe neurological damage caused by post-infectious inflammatory response syndrome (PIIRS). This inflammatory response appears to occur after adequate microbiological control with negative cerebrospinal fluid (CSF) cultures, suggesting that the release of cryptococcal antigen triggers propagation of cryptococcal-antigen-specific CD4 and CD8 T cells in the central nervous system (CNS) with a predominant Th1 response. The increased secretion of cytokines and chemokines, including IFNγ, IL-6, IL-18, and CXCL10, in turn, leads to inflammation and cerebral edema (4). Existing literature shows that IL-6 plays a critical role in inflammation by activating the production of acute phase proteins and stimulating antibody production and CD8 T-cell differentiation (4). IL-6 also skews CD4 T-cell differentiation toward a Th17 response that has been found to be involved in dysregulation of host resistance leading to excessive and chronic inflammation (5, 6). With evidence of dampening this immune-mediated inflammation, high-dose corticosteroids have been successfully employed with improvement in neurological outcomes in patients with PIIRS (7–9).

Further to this inflammatory dysregulation, there is evidence of M2-activated macrophages in the infiltrates of the CNS tissue found in brain biopsies of patients with CM. This M2 skewing is non-protective with suppressed local responses, leading to decreased secretion of TNFα, a key cytokine in resolving fungal infections (7). While HIV-positive and transplant patients are susceptible to CM due to immunosuppression and, in particular, due to lowered T-cell populations, this M2 macrophage bias may suggest a distinct mechanism of immune dysregulation leading to susceptibility in an otherwise healthy individual.

There has also been significant work done in detecting biomarkers of CM inflammation. CSF levels of neurofilament light chain (NFL), a marker of axonal damage, have been shown to predict choroid plexitis on brain MRI, while CSF levels of sCD27, a marker of intrathecal T-cell-mediated inflammation, predict imaging findings of ependymitis (10). Increased levels of these CSF biomarkers have been correlated to worse neurological symptoms (9). Furthermore, they decrease in response to high-dose corticosteroids with corresponding clinical improvement (9).

## Case Presentation

A 15-year-old, previously healthy male, presented for evaluation of a 4-week history of nausea, vomiting, weight loss, and imbalance, along with a 3-week history of vision changes (blurry and double vision). He also described intermittent headache with progressive neck stiffness, photophobia, and tactile temperatures. Examination was notable for meningismus and right lateral rectus nerve palsy. Magnetic resonance imaging (MRI) of the brain noted no intracranial hemorrhage or parenchymal mass, but did demonstrate abnormal FLAIR (fluid-attenuated inversion recovery) signal scattered throughout multiple cerebral sulci along with punctate foci of abnormal FLAIR signal within subcortical white matter associated with subtle leptomeningeal enhancement. Initial CSF studies demonstrated elevated opening pressure, lymphocyte-predominant pleocytosis, low glucose, and elevated protein. *Cryptococcus gattii* was identified on multiplex PCR (and confirmed on fungal culture of CSF fluid), and therapy was initiated with intravenous liposomal amphotericin B (5 mg/kg/day) and oral flucytosine. The patient received serial therapeutic lumbar punctures during his hospitalization to help relieve nausea and headaches caused by increased intracranial pressure. Approximately 10 days after initiation of antifungal therapy, he exhibited neurological changes with increased somnolence, anisocoria, and new fevers. CSF, which was notably PCR and culture negative for the infectious organism at that time, was more aggressively drained to lessen the presumed effects of increased intracranial pressure. Two days later, he became increasingly agitated with worsening mental status; dexamethasone was initiated at 0.3 mg/kg/day due to suspected PIIRS. He defervesced the following day, and his neurological exam began to gradually improve with resolution of agitation and anisocoria. Five days after initiation of the steroid burst, he had completely returned to baseline mental status. He continued a slow IV taper over a 2-week period with transition to oral steroids to complete a planned wean for the same duration of his antifungal therapy.

**Figure 1:**
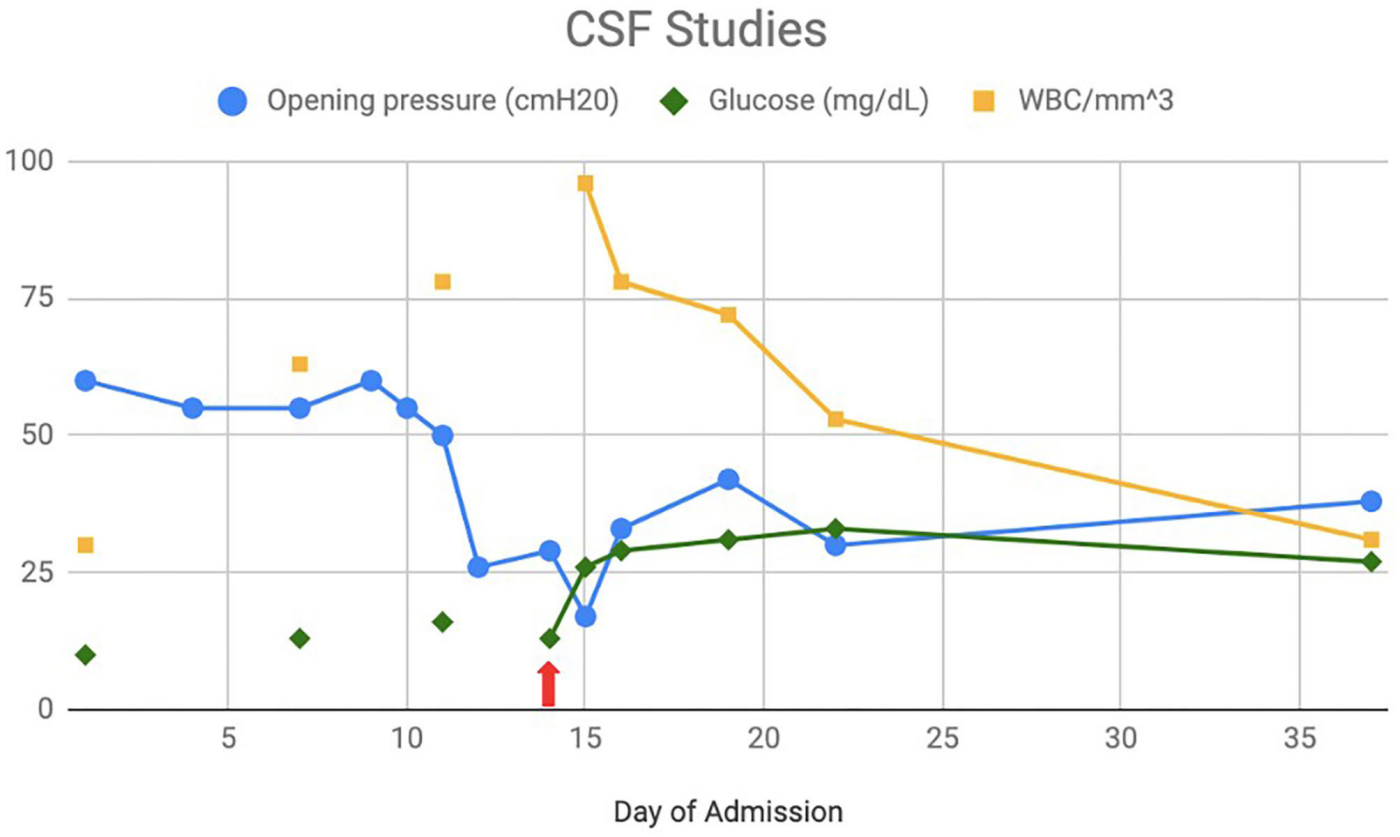
Cerebrospinal fluid (CSF) studies: CSF opening pressure, glucose, and WBC count over the course of initial hospitalization with initiation of steroids on day 13 (red arrow).

As CSF biomarkers were not readily available with practical turnaround times, CSF glucose was initially used to track neurologic recovery during PIIRS (Figure 1) as higher levels have been shown to correspond with more favorable outcomes (11). In addition, CSF cytokines were intermittently tracked over time to monitor changes that might mirror his clinical condition (Table 1). CSF cytokine levels when he first developed PIIRS were significant for elevated sIL-2R, IL-6, and IL-8 with normal IL-1beta, IL-2, IL-4, IL-5, IL-10, IL-12, IL-13, IL-17, IFN-gamma, and TNF-alpha. Repeat cytokine levels 9 days after beginning steroids (with adequate antimicrobial treatment) demonstrated continued elevations of IL-6 (which had decreased markedly) and IL-8 (which remained mildly increased throughout his treatment).

**Table 1:** Cerebrospinal fluid (CSF) cytokine levels over time, during symptomatic periods.

	**Admission**	**Admission**	**Relapse #1**	**Relapse #1**	**Relapse #2**	**Completion**
	**Day 14: After meningitis diagnosis, altered mental status**	**Day 23: 9 days after starting steroids**	**9 months from diagnosis: Weaning steroids, had right-sided symptoms with MRI findings**	**9 months from diagnosis: 3 days after restarting steroids**	**21 months from diagnosis: 4 months after steroid wean, with left-sided symptoms correlated with MRI findings**	**33 months from diagnosis: 3 months after completion of slow steroid taper asymptomatic with normal MRI**
IL-2R (CD25), ref < = 1,033 pg/ml	**9,590**	758	546	449	184	47
IL-6, ref < = 5 pg/ml	**7,480**	**52**	**141**	**31**	**94**	**15**
IL-8, ref < = 5 pg/m.	**196**	**244**	**54**	**138**	**108**	**105**

*Bold values indicate elevation above normal range*.

After stabilization of his condition, and completion of 6 weeks of intravenous liposomal amphotericin B and oral flucytosine, the patient was transitioned to planned fluconazole therapy (for 12-month total antifungal treatment) and discharged on a continued steroid wean with close neurologic and ophthalmologic monitoring. Nine months later, while still on maintenance antifungal therapy and toward the end of his steroid wean, the patient was re-admitted to the hospital after developing right-sided numbness. Magnetic resonance imaging of the brain demonstrated new leptomeningeal enhancement in the left parietal lobe. CSF studies were negative for *Cryptococcus* and other infectious etiologies, while CSF cytokine levels showed notably elevated levels of IL-6 (with mild IL-8 persistence) and normal sIL-2R levels (Table 1). His findings were felt to be due to a relapse of PIIRS rather than infection, and he was restarted on higher doses of steroids with a slower wean over the next 8 months. After receiving a steroid burst of 2 mg/kg for 5 days, IL-6 notably decreased again, while IL-8 remained elevated (as previously), and he was able to taper off slowly after many months without incident. Approximately 4 months after completion of his second steroid wean (and without current antifungal treatment), he developed new left-sided paresthesia and weakness of his upper and lower extremities. Evaluation again was negative for infection with MRI revealing new right-sided leptomeningeal enhancement along with mild elevation in IL-6 and persistence of IL-8. He was treated again with a short course of higher-dose steroids followed by a prolonged taper. His evaluation and imaging after completion of this therapy over the following 8 months has now shown an unremarkable MRI and almost complete normalization of his IL-6 level, after 3 months steroid free.

Initial immunologic workup revealed normal IgG, IgG subclasses, IgA, and IgM with slight elevation in IgE. Protective vaccine titers to tetanus, diphtheria, and *Streptococcus pneumoniae* (18/23) were noted. Lymphocyte enumeration was notable for normal numbers of T, B, and NK cells. Mitogen proliferation showed normal responsiveness to PMA, Pokeweed, and Con A. Neutrophil oxidative burst was normal, and Toll-like receptors 1 to 8 showed normal activity. CH50 was normal, and HIV PCR was negative. Anti-GM-CSF and anti-IFNγ autoantibody titers were negative. Primary immunodeficiency genetic panel did not find any pathogenic variants associated with his clinical or immunologic profile.

## Discussion

The CSF inflammatory milieu we found in our patient was consistent with previously reported elevations in sIL-2R, IL-6, and IL-8 in the setting of cryptococcal infection. These levels are consistent with strong Th1 and Th17 responses with little Th2 response (12). Repeat cytokine levels after starting steroids demonstrated normalization of nearly all cytokines except for IL-6 and IL-8. This suggests that it was primarily the Th1 response, which was dampened by the corticosteroids, while the Th17 response seemed to persist (4, 5). Our CSF cytokine studies also showed low TNFα levels, consistent with the M2 skewing of macrophages, which is thought to contribute to poor antigen clearance and persistent immunologic and inflammatory response in patients with PIIRS (7).

Little exists in the literature regarding the long-term outcomes of cryptococcal meningitis and the clinical course of PIIRS. In a study of previously healthy patients who developed cryptococcal meningoencephalitis, patients demonstrated psychomotor, and executive function deficiencies when neuropsychological testing was performed during the late posttreatment period (13). A follow-up of six cases of spinal arachnoiditis due to PIIRS showed a high rate of morbidity and mortality: one death, two patients who required walking assistance, one with residual wide-based gait, and two with normal ambulation (9). More recently, groups have begun to address establishing a consensus of treatment strategies in order to best impact the long-term sequelae of PIIRS. In a series of 15 adult patients with PIIRS who received a course of pulse steroids followed by prolonged taper, the authors demonstrated improvement in both neurologic and radiologic outcomes (14). However, in this context of need for ongoing suppression, the adverse effects associated with long-term corticosteroid use at any age must also be taken into account. Our patient developed mild hypertension during his corticosteroid treatment, which was managed symptomatically and resolved with cessation of therapy. Ophthalmologic tracking has also noted mild increase in ocular pressures during the treatment course as well. Otherwise, he has not had lingering neurologic findings, though he had been placed on empiric antiseizure medications after his most recent admission for a period of time without any issues.

*Cryptococcus gattii*, in comparison with *C. neoformans*, is usually regarded as a pathogen of apparently immunocompetent patients. However, risk factors, including immunodeficiency and subclinical immune defects, have also been found in the affected population (15). The immune evaluation of our patient, looking at both innate and adaptive immune pathways, did not reveal any predisposing risk for his infection, while genetic screening did not target any specific pathway of affectation. Anti-GM-CSF autoantibodies have also been shown to be a risk factor for CM, while anti-IFNγ autoantibodies have been noted to promote dissemination of cryptococcal infection in murine models (16–18). The serum levels of these antibodies of our patient were also negative.

The cytokines found in the CSF of our patient during his relapse may shed light on the initial stages of PIIRS and the long-term effects of steroids on the trajectory of PIIRS. Only IL-6 and IL-8 remained elevated, while sIL-2R, a marker of T-cell activation, normalized after treatment of initial infection. It seems that his IL-6 level correlated with his PIIRS relapses, while his IL-8 simply remained mildly elevated throughout; however, it is unclear whether the lower levels were due to the early evaluation of PIIRS in the period of flare or due to lack of additional association with infection. Prior CSF cytokine analysis of patients diagnosed with PIIRS has noted the elevation of IL-6 and sIL2R with noted decrease upon treatment with pulse steroids as well (14). In our case, the normalized level of sIL-2R after initial treatment may confirm that, in the absence of active (or very recent) cryptococcal infection, T cells do not play as much of a role in the long-term inflammatory response.

The modulating levels of IL-6 present an interesting target for control of ongoing inflammation in a patient who has cleared a cryptococcal infection. IL-6 is known to be associated with inflammation by directly initiating the acute phase response and promoting the differentiation of CD4 and CD8 T-cells (4, 5). IL-6 has been targeted in other models of inflammatory disease, including rheumatoid arthritis and autoimmune encephalitis (19–21). In patients on IL-6 blockade therapy, corticosteroid wean has been generally successful with maintenance of inflammatory disease control (22). Given these findings in other inflammatory conditions, the short-term use of IL-6 blockade in PIIRS may be considered to help hasten the resolution of inflammation and lessen the risk of inflammatory relapse while decreasing the burden of chronic steroid use. In our patient who showed increase in IL-6 during these periods of flare, this is an attractive option for its steroid-sparing effect, though considerations for dose, duration, and endpoint must be reviewed. This inflammatory pathway warrants further investigation in order to have better impact on long-term clinical outcomes in patients with PIIRS.

## Conclusion

This case is one of the first documented pediatric patients with cryptococcal meningitis-induced PIIRS with successful corticosteroid treatment. The investigations of the CSF cytokine milieu correlate well with the pathophysiology of what is currently understood in PIIRS and offers an attractive target for expanding treatment options for this condition. Additionally, the serial measurements of cytokine responses before and after corticosteroid treatment provides additional information about the immunologic and inflammatory mechanisms that are activated with cryptococcal-induced PIIRS in immunocompetent patients.

## Data Availability Statement

The original contributions presented in the study are included in the article/supplementary material, further inquiries can be directed to the corresponding authors.

## Ethics Statement

Written informed consent was obtained from the minor(s)' legal guardian/next of kin for the publication of any potentially identifiable images or data included in this article.

## Author Contributions

JC and VD took part in patient care, conceptualized, and wrote this manuscript. RC took part in patient care and contributed sections to this manuscript. PW conceptualized and contributed figures and analysis to this manuscript. All authors contributed to manuscript revision, read, and approved the submitted version.

## Conflict of Interest

The authors declare that the research was conducted in the absence of any commercial or financial relationships that could be construed as a potential conflict of interest.

## Publisher's Note

All claims expressed in this article are solely those of the authors and do not necessarily represent those of their affiliated organizations, or those of the publisher, the editors and the reviewers. Any product that may be evaluated in this article, or claim that may be made by its manufacturer, is not guaranteed or endorsed by the publisher.
